# Deoxyguanosine kinase mutation F180S is associated with a lean phenotype in mice

**DOI:** 10.1038/s41366-023-01262-z

**Published:** 2023-01-28

**Authors:** Cédric Francis Borreguero, Stephan Wueest, Constanze Hantel, Holger Schneider, Daniel Konrad, Felix Beuschlein, Ariadni Spyroglou

**Affiliations:** 1 Klinik für Endokrinologie, Diabetologie und Klinische Ernährung, University Hospital Zurich (USZ), University of Zurich (UZH), Zurich, Switzerland; 2 Division of Pediatric Endocrinology and Diabetology, University Children’s Hospital, University of Zurich, Zurich, Switzerland; 3 Children’s Research Center, University Children’s Hospital, University of Zurich, Zurich, Switzerland; 4 Medizinische Klinik und Poliklinik III, University Hospital Carl Gustav Carus Dresden, Dresden, Germany; 5 Medizinische Klinik und Poliklinik IV, Ludwig-Maximilians-University (LMU), Munich, Germany

**Keywords:** Endocrine system and metabolic diseases, Endocrinology

## Abstract

**Background:**

Deoxyguanosine kinase (DGUOK) deficiency is one of the genetic causes of mitochondrial DNA depletion syndrome (MDDS) in humans, leading to the hepatocerebral or the isolated hepatic form of MDDS. Mouse models are helpful tools for the improvement of understanding of the pathophysiology of diseases and offer the opportunity to examine new therapeutic options.

**Methods:**

Herein, we describe the generation and metabolic characterization of a mouse line carrying a homozygous *Dguok*^*F180S/F180S*^ mutation derived from an *N*-ethyl-*N*-nitrosourea-mutagenesis screen. Energy expenditure (EE), oxygen consumption (VO_2_) and carbon dioxide production (VCO_2_) were assessed in metabolic cages. LC-MS/MS was used to quantify plasma adrenal steroids. Plasma insulin and leptin levels were quantified with commercially available assay kits.

**Results:**

Mutant animals displayed significantly lower body weights and reduced inguinal fat pad mass, in comparison to unaffected littermates. Biochemically, they were characterized by significantly lower blood glucose levels, accompanied by significantly lower insulin, total cholesterol, high density lipoprotein and triglyceride levels. They also displayed an almost 2-fold increase in transaminases. Moreover, absolute EE was comparable in mutant and control mice, but EE in mutants was uncoupled from their body weights. Histological examination of inguinal white adipose tissue (WAT) revealed adipocytes with multilocular fat droplets reminiscent of WAT browning. In addition, mRNA and protein expression of *Ucp1* was increased. Mutant mice also presented differing mitochondrial DNA content in various tissues and altered metabolic activity in mitochondria, but no further phenotypical or behavioral abnormalities. Preliminary data imply normal survival of *Dguok*^*F180S/F180S*^ mutant animals.

**Conclusion:**

Taken together, DGUOK mutation F180S leads to a lean phenotype, with lower glucose, insulin, and lipid levels rendering this mouse model not only useful for the study of MDDS forms but also for deciphering mechanisms resulting in a lean phenotype.

## Introduction

Mitochondrial DNA encodes a restricted number of genes including subunits of the triphosphate adenosine (ATP) synthase, cytochrome oxidase and NADH dehydrogenase, all involved in the oxidative phosphorylation and ATP production [1]. Mitochondrial DNA synthesis, itself, relies on nuclear genes. De novo synthesis of deoxyribonucleotides (dNTPs) occurs during nuclear DNA replication, but in resting cells, dNTP supply depends on a rescue pathway, with sequential phosphorylation of deoxyribonucleotides catalyzed by thymidine kinase 1 (TK1) and deoxycytidine kinase (DCK), acting in the cytosol and thymidine kinase 2 (TK2) and deoxyguanosine kinase (DGK or DGUOK), localized in the mitochondria [2]. When the activity of these kinases is reduced or absent, the limited availability of dNTPs leads to mitochondrial DNA depletion syndromes (MDDS). While this group of autosomal recessive disorders has as common root with the reduction of mitochondrial DNA, clinically they present with a wide range of symptoms caused by any combination of hepatopathy, myopathy and encephalopathy, also depending on the specific genetic alteration [3].

Along the same line, *DGUOK* mutations can present in two different forms: one characterized by neonatal-onset hepatopathy and encephalopathy and one by isolated liver disease. In the early-onset form, symptoms appear within the first weeks of life with lactic acidosis and hypoglycemia and progressive liver failure with hepatomegaly, elevated transaminases, cholestasis, and jaundice along with hypotonia, nystagmus and psychomotor retardation [4]. The prognosis of this form is poor, with the majority of affected individuals dying before the age of four [5]. Patients affected by the later-onset isolated form of hepatic disease present in infancy with progressive liver failure with an overall less severe phenotype. However, at later stages patients can develop hepatocellular carcinoma [6, 7]. Remission of hepatic disease has been reported in a single case [8, 9]. Currently, no satisfactory therapy is available for this MDDS. Liver transplantation remains an option but does not seem to improve survival in patients with liver failure and should be considered only in the absence of neurological features [5, 10]. Several *DGUOK* mutations have been identified in patients with hepatocerebral or isolated hepatic form of the disease. No clear genotype-phenotype correlation could be established; however, missense mutations seem to result into a later onset of symptoms and slower disease progression, in comparison to frameshift or non-sense mutations [11]. Furthermore, residual DGUOK activity seems to play an important role in disease progression [12]. Several frameshift or non-sense and missense mutations (e.g., M1I, M1V, S52F, E44K, K51Q, R105*, S107P, E165V, Q170R, W178X, Y191C, H226R, L248P, L250S, F256*) have been reported in patients with DGUOK deficiency [7, 11, 13–16], addressing the need for various in vitro and in vivo models to improve understanding of the varying clinical characteristics of this disease.

Murine models serve as a helpful tool to understand pathophysiology of human disease, allow detailed phenotyping, and can provide the basis for the development of new therapeutic strategies. So far, two Dguok animal models have been described: a complete Dguok knock-out (KO) rat model which did not display a pathological phenotype [17] and a complete murine Dguok KO model, indicative of a hepatic phenotype [18]. Herein, we report the generation and metabolic characterization of a Dguok mutant mouse line, carrying a phenylalanine (F) to serine (S) substitution of residue 180 (*Dguok*^*F180S/F180S*^), leading to a mild form of MDDS.

## Methods

### Generation of the Dguok^F180S/F180S^ mouse line

Our previous research has focused on the identification of mouse lines with hyperaldosteronism using an *N*-ethyl-*N*-nitrosourea (ENU)-mutagenesis screen. This alkylating agent induces point mutations in the spermatogonial stem cells of treated mice. Following this procedure, among others, we established a mouse line of familial hyperaldosteronism, carrying four different point mutations all located on chromosome 6 [19, 20]. By applying an iterative breeding strategy, we were able to segregate the potential candidate genes and established four different mouse lines, each one carrying only one single point mutation. For the *Dguok*^*F180S/F180S*^ (c.539 A > G) mouse line described herein, LC-MS/MS was used to quantify plasma adrenal steroids and, thereby, no significantly elevated aldosterone values could be documented in mutant animals (Supplementary Fig. 1). While the mouse line carrying this specific Dguok mutation was not the appropriate for pursuing the aldosterone driven phenotype, we observed significant weight alterations in genetically affected animals, deserving further metabolic characterization.

More specifically, for the generation of this mouse line, sperm of the previously reported mouse line, archived at the European Mouse Mutant Archive – EMMA, was used for embryo transfer [19, 20]. Revitalized mice, with C3HeB/FeJ background, were imported from Munich Helmholtz Center, Institute for Experimental Genetics. Heterozygous mice carrying the *Dguok*^*F180S/F180S*^ mutation were cross-bred to obtain homozygous mice, together with wild types, used herein as controls, and heterozygous animals, from the same breeding.

### Genotyping

Genotyping of the *Dguok*^*F180S/F180S*^ mouse line was performed with RT-qPCR probes containing Locked Nucleic Acids (LNA), designed to be specific for either the wild-type (WT) or the mutant sequence (TIB Molbiol, Berlin, Germany, Supplementary Table 1). Genotyping was also confirmed with Sanger sequencing of selected samples, performed at Microsynth AG, Balgach, Switzerland).

### Blood and organ sampling

Mice were housed in groups of 2–5 in IVC cages in a controlled environment (20 °C) on a 12 h light/dark photoperiod, in the Laboratory Animal Service Center of the University of Zurich. All animal experiments were approved by the Zurich cantonal authorities (License number 090/2019). Animals were fed normal chow diet (KLIBA NAFAG 3436: 18.5% protein, 4.5% fat, 4.5% fiber, 6.5% ash, 54% NFE) *ad libitum* and had free access to water. To minimize the effect of the circadian rhythm on hormones level, all mice were euthanized between 9 h and 11 h AM. Mice were killed by decapitation under isoflurane anesthesia at the age of 16 weeks. Trunk blood was collected in empty tubes for serum measurements or EDTA coated tubes for plasma measurements. The blood was then centrifuged at 10.000 *g* for 10 min at room temperature and the supernatant was separated and directly frozen at −20 °C.

Brain, liver, and bilateral inguinal fat pads, subscapular brown adipose tissue (BAT) and adrenal glands were prepared and resected. Adrenal glands were cleaned from adjacent fat tissue. Livers and right sided bilateral organs were stored in paraformaldehyde solution (PFA) for further histological analyses. Brains, livers and left sided bilateral organs were snap frozen in liquid nitrogen and stored at −80 °C for further uses.

### Glucose, lipid, electrolytes, and hormonal measurements

Blood glucose levels were determined using the Accu-Chek Aviva glucose meter (Roche, Basel, Switzerland). Serum electrolytes (sodium, potassium and chloride were quantified with the Stat Profile Prime Electrolyte Analyzer (Nova Biomedical, Zürich, Switzerland). Total cholesterol, high density lipoprotein, triglycerides were measured with the AU480 Clinical Chemistry System (Beckman Coulter, Indianapolis, USA). Plasma insulin and leptin levels were quantified with following commercially available assay kits: Mouse insulin ELISA Kit (Mercodia, Uppsala, Sweden), Mouse leptin ELISA kit (Sigma-Aldrich, St. Louis, MO, USA), according to the respective manufacturer’s instructions.

Liquid Chromatography/Mass Spectrometry (LC/MS) for adrenal steroids was performed in EDTA plasma samples in collaboration with the University Hospital Carl Gustav Carus at TU Dresden, Institute of Clinical Chemistry and Laboratory Medicine, Experimental Mass Spectrometry and Trace Elements Lab (Dr. Mirko Peitzsch). The method for analysis of the plasma steroid panel, including validation and assay performance characteristics has been described in detail elsewhere [21].

### Metabolic cage analysis

15-week-old male mice were placed individually in air-tight cages designed for metabolic phenotyping in an open-circuit indirect calorimetric system (PhenoMaster, TSE Systems, Bad Homburg, Germany) for four days as previously described [22]. The average of days 3 and 4 was used for data calculation, since body weight was stable during these two days. A total of 72 data points for food intake, O_2_ consumption, and CO_2_ production were recorded over both 24 h periods. Locomotor activity was measured using a 2-dimensional infrared light-beam. Energy expenditure (EE), oxygen consumption (VO_2_) and carbon dioxide production (VCO_2_) were calculated using the manufacturer’s software and values were additionally corrected for lean body mass (LBM). LBM was calculated according to manufacturer’s software as body weight raised to the power of 0.75. Body weight was daily updated in the software ensuring that EE data normalized to LBM were always taking into account the current body weight of the mice.

### Fecal bomb calorimetry

Two animals of the same genotype were housed together in a clean IVC cage. After 24 h, 1 g of feces were sampled and frozen at −20 °C until analysis. Samples were then dried under a ventilated hood overnight and placed in the decomposition vessel. The decomposition vessel was then placed in the calorimeter bomb and the samples were processed in adiabatic mode. This analysis was performed by the center of Phenogenomics of the École Polytechnique Fédérale in Lausanne.

### Real-time PCR

Whole organs were homogenized in the RNA lysis buffer (Zymo Research, Irvine, CA, USA) using a shaking homogenizer with ceramic beads (MP Biomedical INC, Illkirch, France). Upon 5 min centrifugation at 10.000 *g* the clean supernatant was used for RNA extraction. For adipose tissue, the upper phase containing fat was discarded and not used. Upon extraction, the concentration of RNA was measured with NanoDrop One UV Spectrophotometer (ThermoFisher Scientific, Waltham, MA, USA). 2 µg of RNA was converted to cDNA using High-Capacity cDNA Reverse Transcription kit with RNase Inhibitor (Applied Biosystems, Waltham, MA, USA). The cDNA was finally diluted to a concentration of 5 ng/μL. The SsoFast EVAGreen Supermix Mastermix was used to quantify the investigated genes. For the gene expression analysis, 10 ng of cDNA was pipetted to the Mastermix. The Cycle program in the Quant 5 was 5’ @ 95 °C then 40 cycles with following conditions: 15”@95 °C and 30”@60 °C. To ensure the absence of unspecific product, a melting curve analysis between 55 °C and 95 °C was also performed at the end. Quantification of gene expression was adjusted using the Tbp mouse gene expression for the investigated adipose tissues and using Gapdh as housekeeping gene for all other tissues.

### Western blotting

Acrylamide gels were cast one day before running the gel or at the same day using standard procedures. Whole organs were homogenized using a shaking homogenizer with 4 mm ceramic beads in RIPA Buffer with protease inhibitor (cOmplete mini, Roche, Basel, Switzerland) and then centrifuged. The protein concentration was measured with the Pierce BCA Protein Assay kit (ThermoFisher, Waltham, MA, USA) and the absorption measured at 565 nm. Protein was then dissolved to the desired concentration using RIPA buffer with Proteinase inhibitor and 2x loading buffer with DTT (Roche, Basel, Switzerland). For the inguinal fat pad 30 μg of protein were loaded. For the other organs, 7.5 μg of protein were loaded. Proteins were transferred to a Nitrocellulose membrane (GE Healthcare, Chicago, IL, USA) with a Blot transfer machine (Bio-Rad Laboratories, Hercules, CA, USA). The membrane was then blocked with a blocking buffer containing 5% dry milk. Next, the membrane was incubated overnight at 4 °C with the primary antibody (dilution 1:1000). The membrane was washed 3 times and then incubated 1 h at RT with the secondary antibody (dilution 1:10000). Finally, after further washing (3x), the membrane was incubated with the detection reagent (West Pico PLUS, ThermoFisher, Waltham, MA, USA) and measured with the Western Blot Imaging System (Fujifilm, Tokyo, Japan). Primary antibodies (UCP1 polyclonal antibody (PA5-29575, ThermoFisher, Waltham, MA, USA), GAPDH - D16H11 Rabbit mAb (Cell Signaling Technology, Danver, MA, USA)) were diluted at 1:1000, while secondary antibodies were used at 1:10000 concentration (Rabbit HRP linked, NA9340, Merck, Darmstadt, Germany).

### Histological analysis

Tissues remained in 4% paraformaldehyde overnight and then were dehydrated, embedded in paraffin, sectioned, and stained with hematoxylin and eosin following standard protocols. Hematoxylin and eosin (H/E)-stained adrenal sections were examined with a light microscope using magnifications of ×40 and ×400. For immunohistochemical staining for Ki-67 paraffin-embedded sections were rehydrated, heated in EDTA 1 mM, pH 9.0, SDS 0.05% in the microwave for antigen retrieval, blocked with 3% H2O2 in methanol for 10 min, and incubated with blocking buffer for 15 min. Ki-67 was immunolocalized overnight at 4 °C by means of a rabbit monoclonal antibody (RM9106-s Thermofisher, MA, USA) in a dilution of 1:300 in blocking buffer. After rinsing for 15 min in PBS, SignalStain® Boost IHC Detection Reagent (HRP, Rabbit, CellSignal, MA, USA) and Sigma Fast DAB (Sigma, Munich, Germany) were used for visualization. Transmission electron microscopy of BAT of a *Dguok*^*F180S/F180S*^ mouse and an unaffected littermate was performed in collaboration with the Center for Microscopy and Image Analysis of the University of Zurich (https://www.zmb.uzh.ch/en.html).

### Statistical analysis

Sample size was calculated based on the body weight difference observed between WT and mutant animals of the same sex in a previous pilot experiment (with an alpha error of 0.05 and a power of 0.8) resulting in an n-number of 5 animals per genotype. The *n*-number of samples included in all experiments/results presented herein was at least five per genotype. Animals/samples were included in the analysis depending on their availability after breeding. No randomization was applied. The investigators were not blinded to the group allocation during the experiments. Statistical analysis was carried out with the Prism 3.02 (GraphPad Software). Statistical significance was determined using the unpaired *t*-test for normally distributed parameters and Mann–Whitney test for non-normally distributed parameters. To investigate body mass-dependence of EE, a regression-based analysis-of-covariance (ANCOVA) was performed as previously described [23, 24]. Statistical significance was denoted by asterisks in the figures as ^*^P < 0.05, ^**^P < 0.01 and ^***^P < 0.001.

## Results

### Significantly lower 11-deoxycorticosterone and corticosterone levels in Dguok^F180S/F180S^ male mice

As part of the characterization of the ENU-derived mouse lines, the Dguok^F180S/F180S^ line was examined for its adrenal steroid phenotype by LC-MS/MS. Thereby, no significant differences could be observed in the aldosterone levels of this mouse line (Supplementary Fig. 1, E and J). In contrast, male *Dguok*^*F180S/F180S*^ mutant mice displayed significantly lower 11-deoxycorticosterone and corticosterone levels than their unaffected littermates (Supplementary Fig. 1B, C).

### Reduced body weight and fat mass of Dguok^F180S/F180S^ mice

**Fig. 1:**
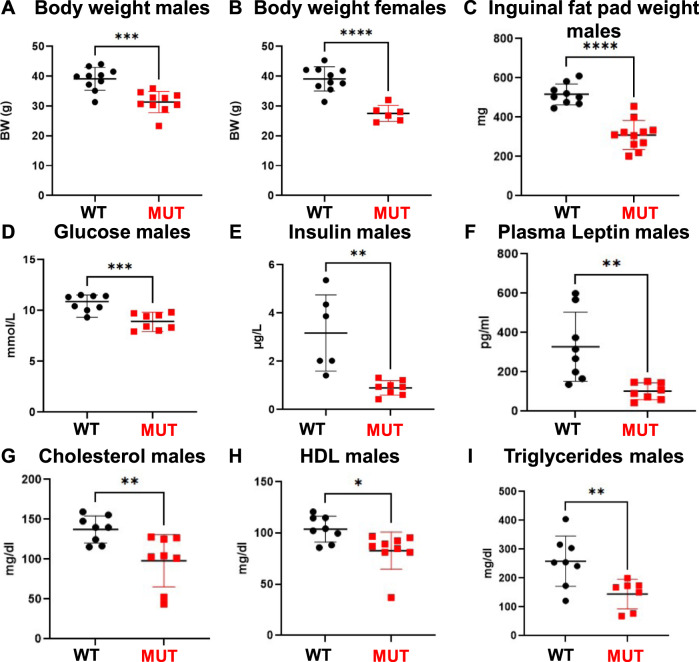
Reduced body weight and fat mass of Dguok^F180S/F180S^ mice. Body weight of male animals (**A**), body weight of female animals (**B**), weight of the inguinal fat pad (**C**), blood glucose levels in male animals (**D**), insulin levels (**E**), plasma leptin levels (**F**), cholesterol (**G**), HDL (**H**) and triglycerides (**I**). WT: Wild type animals, MUT: *Dguok*^*F180S/F180S*^, HDL High density lipoproteins. *p < 0.05, **p < 0.01, ***p < 0.001, ****p < 0.0001 (Student’s *t* test). Values are expressed as mean ± SEM.

On gross observation, *Dguok*^*F180S/F180S*^ mutant animals of both sexes at the age of 16 weeks moved and behaved normally without obvious phenotypic alterations but displayed significantly lower body weights than their unaffected littermates (Fig. 1A, B). For all subsequent analyses we focused on male animals. In line with the previous finding, inguinal fat pads of *Dguok*^*F180S/F180S*^ male mutant animals were significantly lighter compared to controls (Fig. 1, C). While serum sodium, potassium and chloride levels did not differ between controls and Dguok mutant mice (data not shown), blood glucose levels were significantly reduced in *Dguok*^*F180S/F180S*^ mutants (Fig. 1D). Of note, the low blood glucose levels of mutant animals were accompanied by low insulin levels (Fig. 1E). Plasma leptin levels were significantly reduced in Dguok mutant animals (Fig. 1F). Finally, *Dguok*^*F180S/F180S*^ mutant animals presented significantly lower lipid levels, that is, lower total cholesterol, lower HDL cholesterol and lower triglyceride levels (Fig. 1G–I).

### Mild hepatic impairment in Dguok^F180S/F180S^ mice

**Fig. 2:**
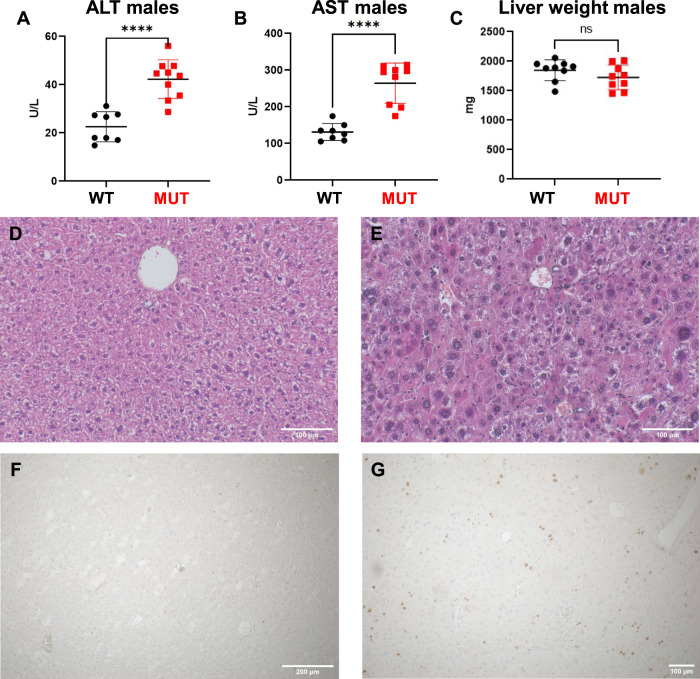
Mild hepatic impairment in Dguok^F180S/F180S^ mice. Comparison of alanine aminotransferase (ALT) (**A**), aspartate aminotransferase (AST) levels (**B**) and of liver weights (**C**). H/E staining of hepatic tissue (**D**, **E**). Ki-67 expression (**F**, **G**). WT: Wild type animals, MUT: *Dguok*^*F180S/F180S*^. *p < 0.05, **p < 0.01, ***p < 0.001, ****p < 0.0001 (Student’s *t* test). Values are expressed as mean ± SEM.

Alanine aminotransferase (ALT) and aspartate aminotransferase (AST) levels of *Dguok*^*F180S/F180S*^ mutant animals were significantly increased in comparison to controls (Fig. 2A, B), however, not exceeding a two-fold increase. Liver weights did not differ between *Dguok*^*F180S/F180S*^ and control mice (Fig. 2C). H/E staining of the hepatic tissue demonstrated larger hepatocytes with larger nuclei in mutant mice, without signs of increased steatosis and mild to absent cholestasis (Fig. 2D, E). The Ki-67 expression in the liver was increased in Dguok mutant animals (Fig. 2F, G), possibly suggesting increased proliferation due to regenerative stress.

### Energy expenditure is uncoupled from body mass in Dguok ^F180S/F180S^ mice

**Fig. 3:**
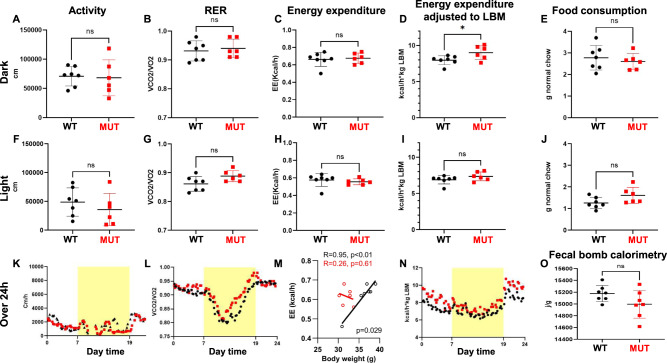
Energy expenditure is uncoupled from body mass in Dguok^F180S/F180S^ mice. Comparison of locomotor activity (**A**, **F**, **K**), RER (**B**, **G**, **L**), absolute energy expenditure (**C**, **H**), energy expenditure adjusted to LBM (**D**, **I**, **N**) and food consumption (**E**, **J**) in the dark (upper panels) and light phase (middle panels) and plotted over a period of 24 h (lower panels). Correlation of energy expenditure to body weight (**M**, black dots: WT, red dots: MUT). Energy excreted measured with fecal bomb calorimetry (**O**). WT: Wild type animals, MUT *Dguok*^*F180S/F180S*^, RER Respiratory exchange ratio, EE energy expenditure. *p < 0.05 (Student’s *t* test). Values are expressed as mean ± SEM.

In order to elaborate on the mechanisms contributing to blunted weight gain in mutant mice (Fig. 1A, B), male animals were placed in metabolic cage units. Thereby, mutant animals did not display any significant difference in their locomotor activity (Fig. 3A, F, K), or food consumption (Fig. 3E, J) compared to unaffected littermates during both the dark and the light phase. Similarly, mean respiratory exchange ratio (RER) did not differ between the two genotypes during dark and light phase (Fig. 3B, G). Still, when observing the RER values plotted over 24 h, an RER of almost 1 was observed towards the end of the light phase (when animals were still asleep) in mutant animals. Such RER of mutant mice was rapidly normalized back to the level of control animals at the beginning of the dark phase, when animals started eating again (Fig. 3L). However, when analyzing the different time points, a statistically significant difference could only be documented in the time frame 14–16 h between mutant and controls (*P* < 0.05, Fig. 3L). Furthermore, although *Dguok*^*F180S/F180S*^ mutant mice did not differ from controls in terms of absolute EE during both the light and dark phase (Fig. 3C, H), *Dguok*^*F180S/F180S*^ mutant mice demonstrated significantly higher EE during the dark phase when EE was normalized to lean body mass (LBM) (Fig. 3D, I, N). When analyzing body mass-dependence of EE using ANCOVA, control but not mutant animals displayed a significant positive correlation, leading to significantly different slopes of the regression lines (Fig. 3M). Finally, to exclude impaired intestinal nutrient absorption capacity as the cause of reduced body weight in mutant mice, nutrient absorption was assessed by fecal bomb calorimetry. As depicted in Fig. 3O, no significant difference was observed in excreted caloric loss between mutant and littermate mice.

### Increased browning of inguinal WAT in Dguok^F180S/F180S^ mice

**Fig. 4:**
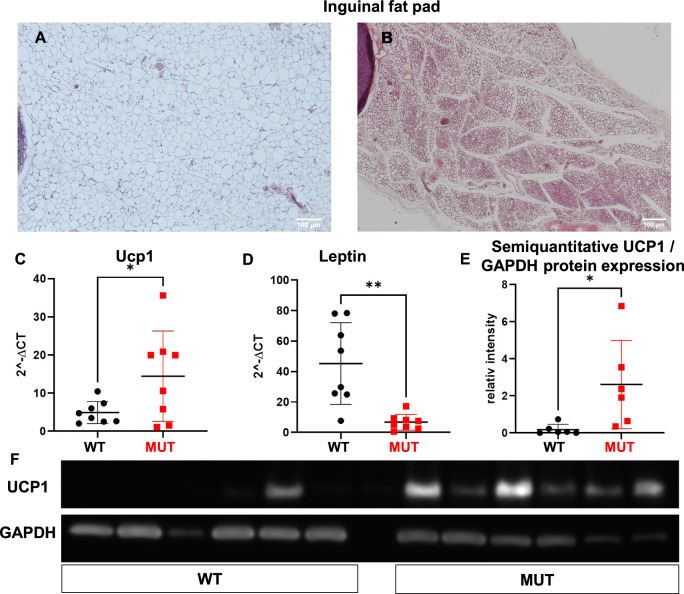
Increased browning of inguinal WAT. Hematoxylin/eosin (H/E) staining of inguinal fat pad of control littermate (**A**) and mutant mice (**B**). mRNA expression of *Ucp1* (**C**) and *leptin* (**D**). Semiquantitative UCP1 to GAPDH protein expression (**E**). Representative western blot of UCP1 protein levels in inguinal WAT (**F**). WT: Wild type animals, MUT: *Dguok*^*F180S/F180S*^, Ucp1: Uncoupling protein 1, GAPDH: Glyceraldehyde-3-Phosphate Dehydrogenase. *p < 0.05, **p < 0.01 (Student’s *t* test). Values are expressed as mean ± SEM.

Increased EE may suggest activation of BAT or browning of WAT in mutant mice. We therefore performed histological analysis of inguinal WAT. As depicted in Fig. 4A, B, mutant mice showed smaller adipocytes, with reduced fat content and multilocular fat droplets reminiscent of WAT browning. Moreover, mutant mice exhibited significantly higher Ucp1 (Fig. 4C), but lower leptin (Fig. 4D) mRNA expression in inguinal fat pads. Similarly, UCP1 protein levels were higher in mutant mice (Fig. 4E, F). In contrast, UCP1 protein levels were similar in subscapular BAT of Dguok mutant and littermate mice (Supplementary Fig. 2A). In addition, brown adipocytes of subscapular BAT were smaller in Dguok mutant compared to littermate mice (Supplementary Fig. 2B, C).

### Altered mRNA expression of mitochondrial enzymes in Dguok^F180S/F180S^ mice

**Fig. 5:**
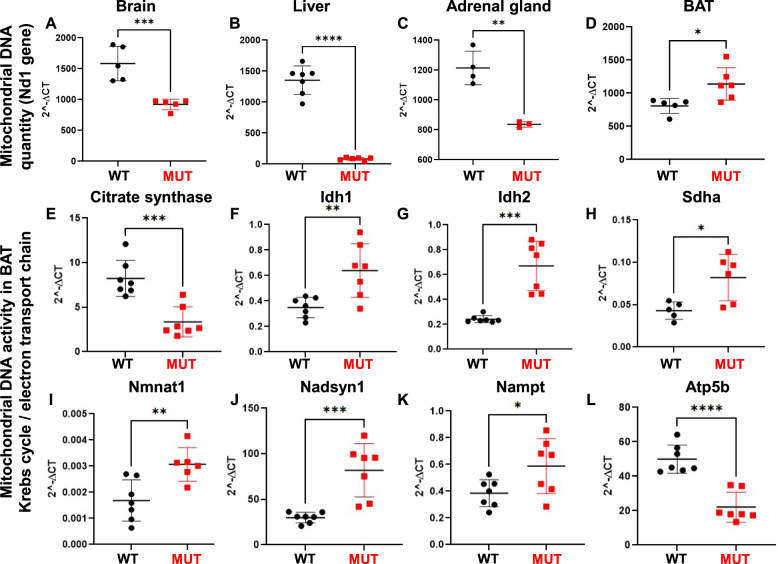
Altered mRNA expression of mitochondrial enzymes in *Dguok*^*F180S/F180S*^ mutant mice. Mitochondrial DNA quantity in brain (**A**), liver (**B**), adrenal gland (**C**) and BAT (**D**). mRNA expression of enzymes involved in the Krebs cycle (*Citrate synthase* (**E**), *Idh1* (**F**), *Idh2* (**G**), *Sdha* (**H**)) and in the electron transport chain (*Nmnat1* (**I**), *Nadsyn1* (**J**), *Nampt* (**K**)), mRNA expression of ATP synthase - *Atp5b* (**L**) in BAT. WT: Wild type animals, MUT: *Dguok*^*F180S/F180S*^, BAT: Brown adipose tissue, Idh1: Isocitrate Dehydrogenase 1, Idh2: Isocitrate Dehydrogenase 2, Sdha: Succinate Dehydrogenase Complex Flavoprotein Subunit A, Nmnat: Nicotinamide mononucleotide adenylyltransferase 1, Nadsyn1: NAD Synthetase 1, Nampt: Nicotinamide Phosphoribosyltransferase, Atp5b: ATP synthase F1 subunit beta. *p < 0.05, **p < 0.01, ***p < 0.001, ****p < 0.0001 (Student’s *t* test). Values are expressed as mean ± SEM.

As DGUOK plays an important role in mitochondrial function, mice were characterized for possible mitochondrial alterations. Mitochondrial DNA quantification in various tissues/organs displayed significantly lower mitochondrial DNA levels in brain, liver, and adrenal glands, but significantly higher DNA level in BAT of *Dguok*^*F180S/F180S*^ mutant mice (Fig. 5A–D). We next determined mRNA expression of enzymes involved in the Krebs cycle and the electron transport chain. As depicted in Fig. 5E, expression of *citrate synthase*, which catalyzes the first step providing substrate to the Krebs cycle, was significantly suppressed in mutant animals. In contrast, expression of *Idh1*, *Idh2* and *Sdha*, all three enzymes catabolizing different steps of the Krebs cycle were significantly higher in Dguok mutant animals (Fig. 5F–H). Similarly, all three enzymes involved in the NAD + biosynthetic pathway were significantly upregulated in Dguok mutant animals (Fig. 5I–K). Moreover, mRNA expression of ATP synthase *Atp5b* was significantly reduced in mutant mice (Fig. 5L).

### Increased mRNA expression of deoxycytidine kinase (DCK) in liver but not BAT of Dguok ^F180S/F180S^ mice

**Fig. 6:**
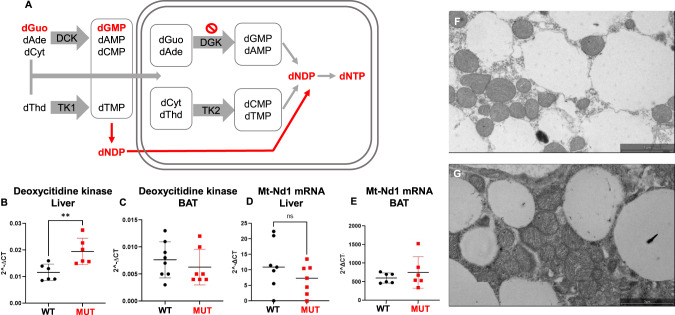
Increased mRNA expression of deoxycytidine kinase (DCK) in liver but not BAT of *Dguok*^*F180S/F180S*^. **A** Simplified schematic representation of the deoxyribonucleotide rescue pathway, with dNDPs generated outside the mitochondria. mRNA expression of the nuclear-encoded gene deoxycytidine kinase (*Dck*) in the liver (**B**) and in BAT (**C**) and of the mitochondrial *Nd1* gene in the liver (**D**) and BAT (**E**) of *Dguok*^*F180S/F180S*^ mutant and control littermate mice. Electron microscopy image of BAT of control (**F**) and *Dguok*^*F180S/F180S*^ mutant (G) mouse (scale bar 3 µm). WT Wild type animals, MUT *Dguok*^*F180S/F180S*^, BAT Brown adipose tissue, dGuo deoxyguanosine, dAde deoxyadenosine, dCyt deoxycytidine, dThd deoxythymidine, DCK deoxycytidine kinase, TK1 Thymidine kinase 1, dGMP deoxyguanosine monophosphate, dAMP: deoxyadenosine monophosphate, dCMP deoxycytidine monophosphate, dTMP deoxythymidine monophosphate, DGK deoxyguanosine kinase, TK2 Thymidine kinase 2, dNDP deoxyribonucleotide diphosphate, dNTP deoxyribonucleotide triphosphate. **p < 0.01 (Student’s *t* test). Values are expressed as mean ± SEM.

Given the mild phenotype observed in this mouse line based on the absence of neurological or hepatic features, we hypothesized on possible compensatory mechanisms. As a rescue pathway for deoxyribonucleoside biosynthesis has been described in the literature, the activity of this pathway was investigated [2]. Specifically, it is appreciated that deoxycytidine kinase (DCK) is able to transform deoxyguanosine to deoxyguanosine monophosphate outside the mitochondria and overcome a relative or absolute DGK absence (Fig. 6A, red labeled pathway). However, in *Dguok*^*F180S/F180S*^ animals, although *Dck* was expressed at higher levels in the liver (Fig. 6B), this was not the case in BAT, with *Dck* expression levels being comparable between the genotypes, suggesting that this rescue pathway is inactive in BAT (Fig. 6C). The mRNA expression of the mitochondrial gene *Nd1* in the liver, BAT (Fig. 6D, E) and in the adrenal gland (data not shown) did not differ between controls and *Dguok*^*F180S/F180S*^ mice, either. *Dguok*^*F180S/F180S*^ mutant animals did not display a significantly different number of mitochondria in their BAT, neither showed any pronounced differences in their size or microscopic structure (Fig. 6F, G).

## Discussion

In summary, the mouse model presented herein carries a homozygous *Dguok* point mutation (F180S) leading to an aberrant metabolic phenotype, characterized by reduced body weight and subcutaneous fat pads, while no other phenotypic abnormalities, such as hepatic or neurological involvement are present that typically characterize MDDS. The mild hepatic impairment documented biochemically and histologically did not seem to phenotypically affect the mice. Thereby, *Dguok*^*F180S/F180S*^ mice extend the spectrum of MDDS with the possibility to specifically characterize metabolic consequences of the disease.

In variance to currently described *DGUOK* animal models, the *Dguok*^*F180S/F180S*^ mutant mouse line provides a metabolic phenotype. Specifically, mutant animals had significantly lower body and inguinal fat pad weights, but no other gross phenotypical abnormalities. Furthermore, *Dguok*^*F180S/F180S*^ mice presented lower blood glucose values, which is a typical initial sign of MDDS due to Dguok deficiency in humans. However, Pronicka et al. describe an islet cell hyperplasia and a hyperinsulinemia in two patients with Dguok deficiency [13], and, similarly, a further case report presents a hyperinsulinemic hypoglycemia due to a homozygous DGUOK Phe256* mutation [25]. Unlike these cases, the *Dguok*^*F180S/F180S*^ animals presented with significantly lower insulin levels than their unaffected littermates, suggestive of a negative feedback mechanism to compensate for the lower glucose levels. In parallel, these mutant mice displayed low corticosterone values, originating from the adrenal glands, that also presented a very low mitochondrial DNA content. As adrenal steroidogenesis is partly dependent on mitochondrial function, Dguok deficiency could contribute to lack of adrenal counter-regulation thereby accentuating the reduction in blood glucose levels. The *Dguok*^*F180S/F180S*^ mouse line also displayed significantly lower cholesterol, HDL and triglyceride levels. The lipid profile of affected individuals with MDDS due to DGUOK deficiency has not been described in detail so far, but the Dguok KO mouse line, previously described, presents an opposed biochemical profile, with significantly increased cholesterol levels in the KO animals [18]. Furthermore, the *Dguok*^*F180S/F180S*^ mice displayed up to two-fold increased transaminase levels (ALT and AST), suggestive of a mild hepatic impairment, whereas the Dguok KO mouse model, previously described, presents with a 4- to 5-fold increase in transaminases. Histological signs of hepatic damage are present at different extent in both mouse models [18].

*Dguok*^*F180S/F180S*^ mutant animals had normal or even increased intestinal absorption, excluding a malabsorption of nutrients as causative for their body weight phenotype. Additionally, they did not differ in their absolute EE from control animals. However, in *Dguok*^*F180S/F180S*^ animals, EE appeared uncoupled from their body mass. The latter may be due to increased browning of white adipose tissue in *Dguok*^*F180S/F180S*^ animals, as reflected by increased UCP1 protein and mRNA levels in inguinal WAT as well as the appearance of multilocular fat cells on histological examination. These mice did not display any neurological abnormalities, possibly due to the lower but maintained at >50% mitochondrial DNA content in the brain. The fact that they presented a mild hepatic pathology, despite the almost nonexistent mitochondrial DNA in their liver can potentially be explained by the increased hepatic expression of deoxycytidine kinase, the key enzyme for the rescue pathway for dNTP synthesis. Another compensatory mechanism is suggested since we observed an unaffected mRNA expression of mitochondrial genes such as Nd1 in *Dguok*^*F180S/F180S*^ animals, despite the low mitochondrial DNA content in this tissue. Surprisingly, these animals present increased mitochondrial DNA in the BAT, with an apparently intact mRNA expression of mitochondrial genes, such as Nd1 in this tissue. It has been previously acknowledged, that mitochondria from different cell types are functionally unique, depending on their respective nuclear background, to address the needs of different cells and BAT is recognized as a tissue with high mitochondrial concentration [26, 27]. Whether the necessity for an increased mitochondrial DNA replication in this tissue serves as a mechanism to escape the mutational effect remains unclear. In line with this concept, electron microscopy revealed no pronounced differences in number, size or structure of mitochondria in the BAT of mutant animals. Still, in spite of the increased mitochondrial DNA in BAT, the expression of various enzymes involved in the Krebs cycle and electron chain transport presented alterations, suggestive of a reduced flow of substrates in the Krebs cycle and a compensatory increased catalyzing of intermediate products. The current mouse model has some phenotypic overlap with the previously described *Dguok* KO model, that is characterized by low body weight and decreased subcutaneous fat layer [18]. Furthermore, this mouse line also presents an altered expression of the enzymes of the Krebs cycle [28].

*Dguok*^*F180S/F180S*^ mice have not yet been systematically observed for the assessment of their life span. According to our preliminary observation, these animals survived up to 30 weeks without further apparent phenotypical or behavioral abnormalities, in line with the preliminary survival estimates of the murine Dguok KO mouse line [18, 28]. The differential mitochondrial DNA content in various tissues might play a role in the lean phenotype presented herein, and it seems, that the *Dguok*^*F180S/F180S*^ mouse line also possesses sufficient compensatory pathways ensuring sufficient mitochondrial DNA levels, that do not further influence their phenotypical appearance and survival.

In MDDS due to *DGUOK* mutations, the phenotypical abnormalities, the time of onset and the course of the disease present with a large variety among genetically affected patients. Both human and mouse Dguok genes contain 277 amino acids and present homology with 75% identities and 85% positive residues [29]. The position F180 is well conserved among species (Supplementary Fig. 3). In the case of F180S substitution, the non-polar, hydrophobic phenylalanine is replaced by a polar and hydrophilic serine, affecting helix propensity, and causing structural changes in the predicted three-dimensional structure of the protein (Supplementary Fig. 4). The previously described W178X mutation in close proximity to the F180S mutation, is associated with a severe and lethal hepato-cerebral form of MDDS in the affected individual [11]. In contrast, as the F180S mutation originates from an ENU mutagenesis screen, this setting might have favored the milder phenotype described herein.

Taken together, we herein describe the generation and metabolic characterization of a *Dguok*^*F180S/F180S*^ mutant mouse line, that displays a lean phenotype, with reduced subcutaneous fat pads, characteristics of WAT browning, and increased EE. Furthermore, mutant animals are characterized by lower blood glucose, insulin, and lipid levels. This mouse line presents differential mitochondrial DNA quantities in various tissues and altered metabolic function in the mitochondria, but no further phenotypical abnormalities observed in MDDS forms. These data are suggestive of the presence of compensatory mechanisms in the context of this specific mutation, ensuring sufficient mitochondrial DNA levels that do not further influence phenotype and survival. This mouse model could serve in the future not only in the study of MDDS forms but also in the understanding of mechanisms resulting in a lean phenotype.

## Supplementary information

Supplemental Material

## Data Availability

The datasets generated and analysed during the current study are available from the corresponding author on reasonable request.
