# Supplementary material for: Deoxyguanosine kinase mutation F180S is associated with a lean phenotype in mice
Source: Int J Obes (Lond). 2023 Jan 28;47(3):215–23. doi: 10.1038/s41366-023-01262-z (PMC10023562; doi:10.1038/s41366-023-01262-z)
Supplement: Supplementary file 1 — Supplemental Material [file 41366_2023_1262_MOESM1_ESM.docx]

**Deoxyguanosine kinase mutation F180S is associated with a lean phenotype in mice.**

**Supplemental Figure 1 Significantly lower 11-deoxycorticosterone and corticosterone levels in Dguok ^F180S/F180S^ mice.**

Progesterone (A and F), 11-Deoxycorticosterone (B and G), Corticosterone (C and H), 18-OH Corticosterone (D and I) and Aldosterone (E and J) levels of male (upper panel) and female (lower panel) *Dguok^F180S/F180S^* mice and their unaffected littermates. WT: Wild type animals, MUT: *Dguok^F180S/F180S^*. *p<0.05 (Student’s *t* test). Values are expressed as mean ± SEM.


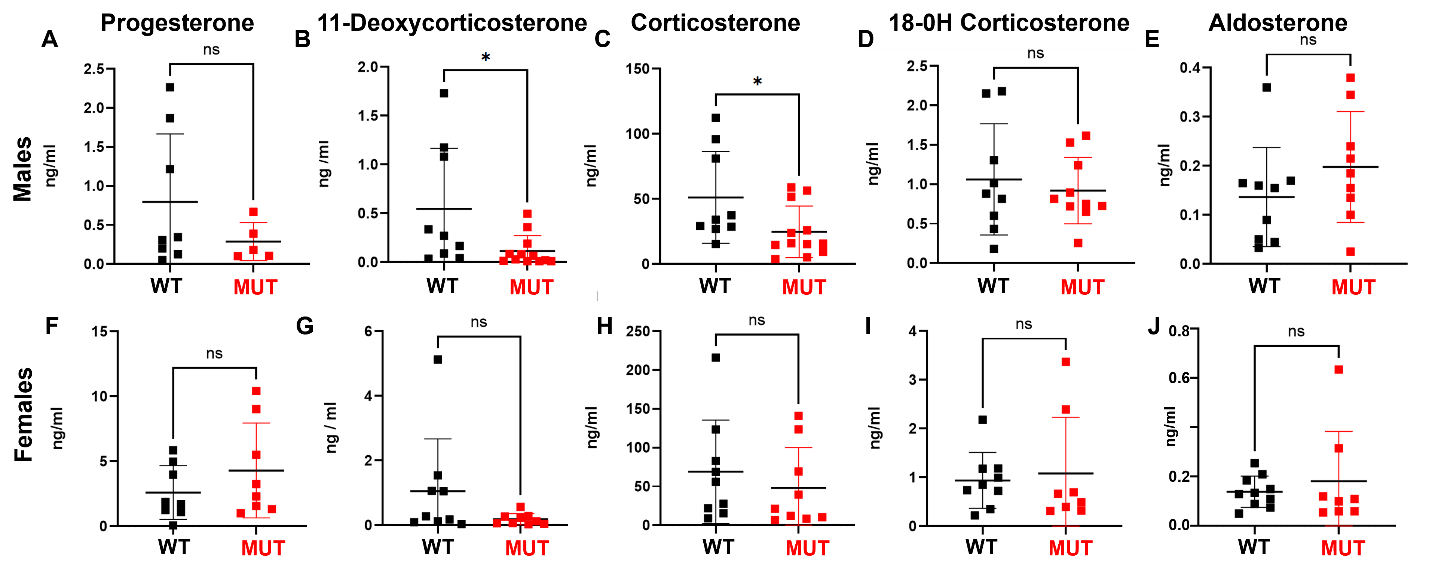


**Supplemental Figure 2 Similar UCP1 protein levels in *Dguok^F180S/F180S^* mutant and control littermate mice in subscapular brown adipose tissue.**

Representative western blot of UCP1 protein levels in BAT (A). H/E staining of the BAT of control littermate (B) and mutant animals (C). WT: Wild type animals, MUT: *Dguok^F180S/F180S^*, Ucp1: Uncoupling protein 1.


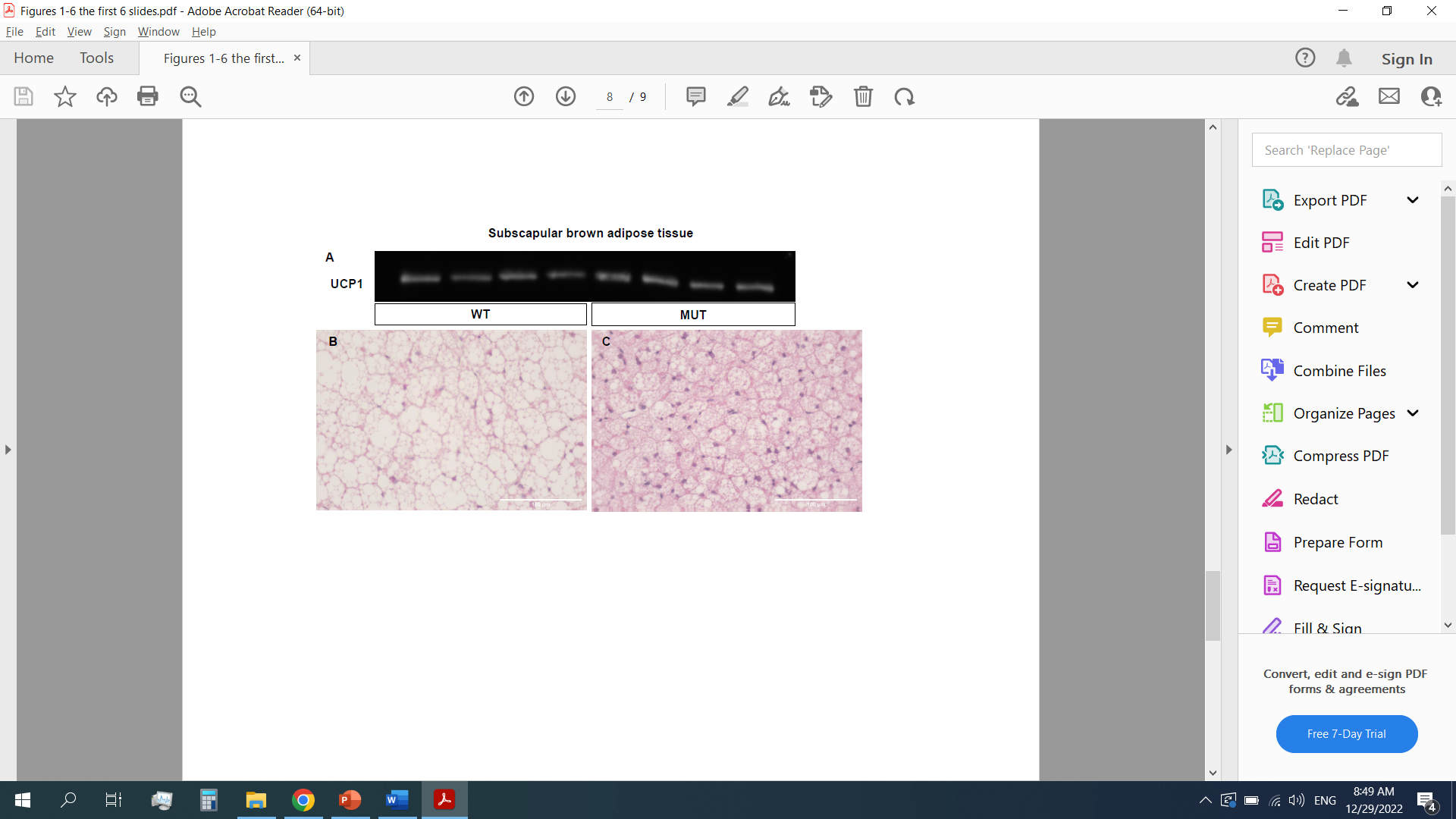


**Supplemental Figure 3 Conservation of the missense mutation F180S among species.**


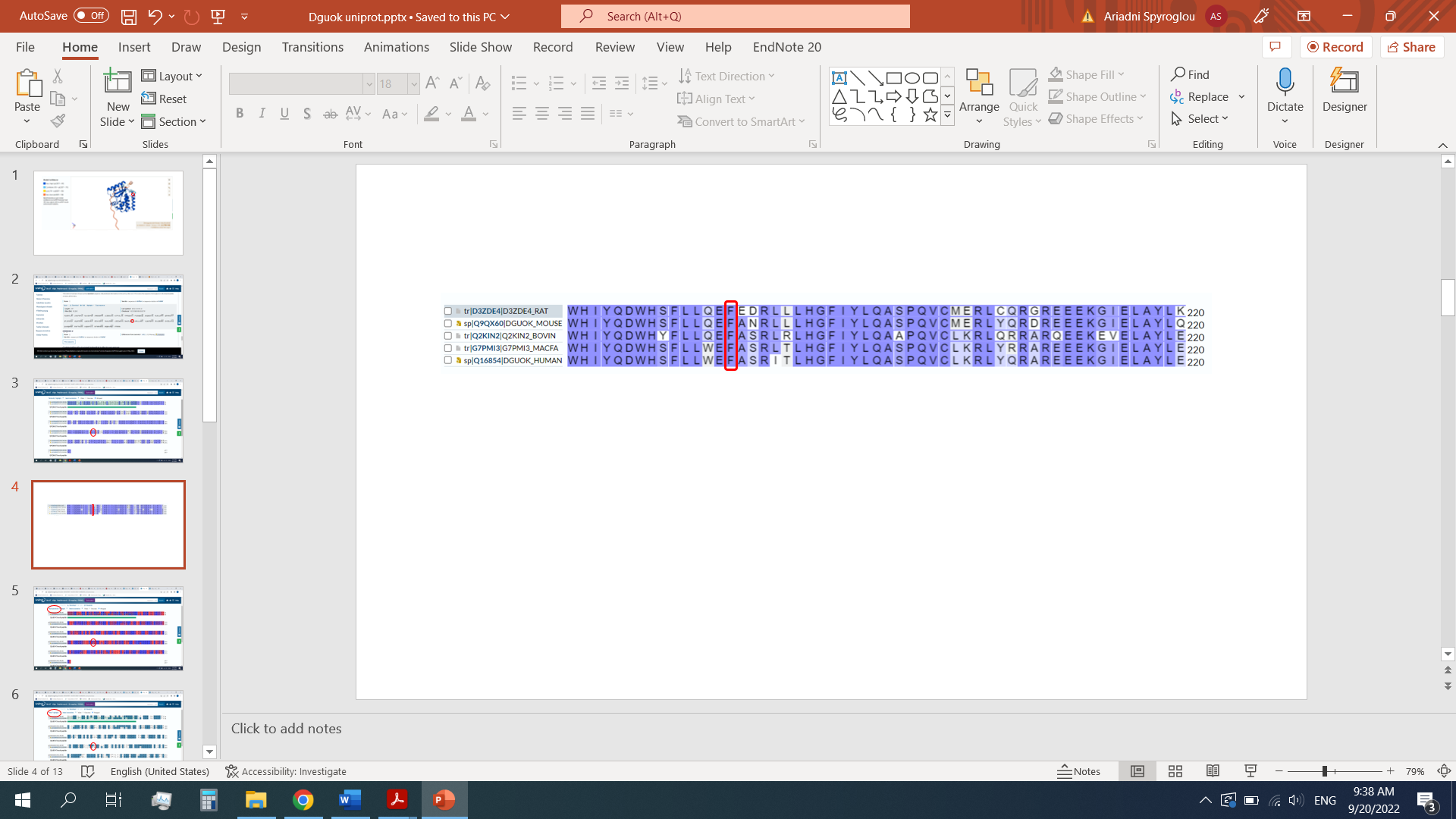


**Supplemental Figure 4: Top 5 predicted 3-dimensional models for the wild type (WT) and F180S mutant DGUOK protein (Source https://zhanggroup.org/I-TASSER/).**


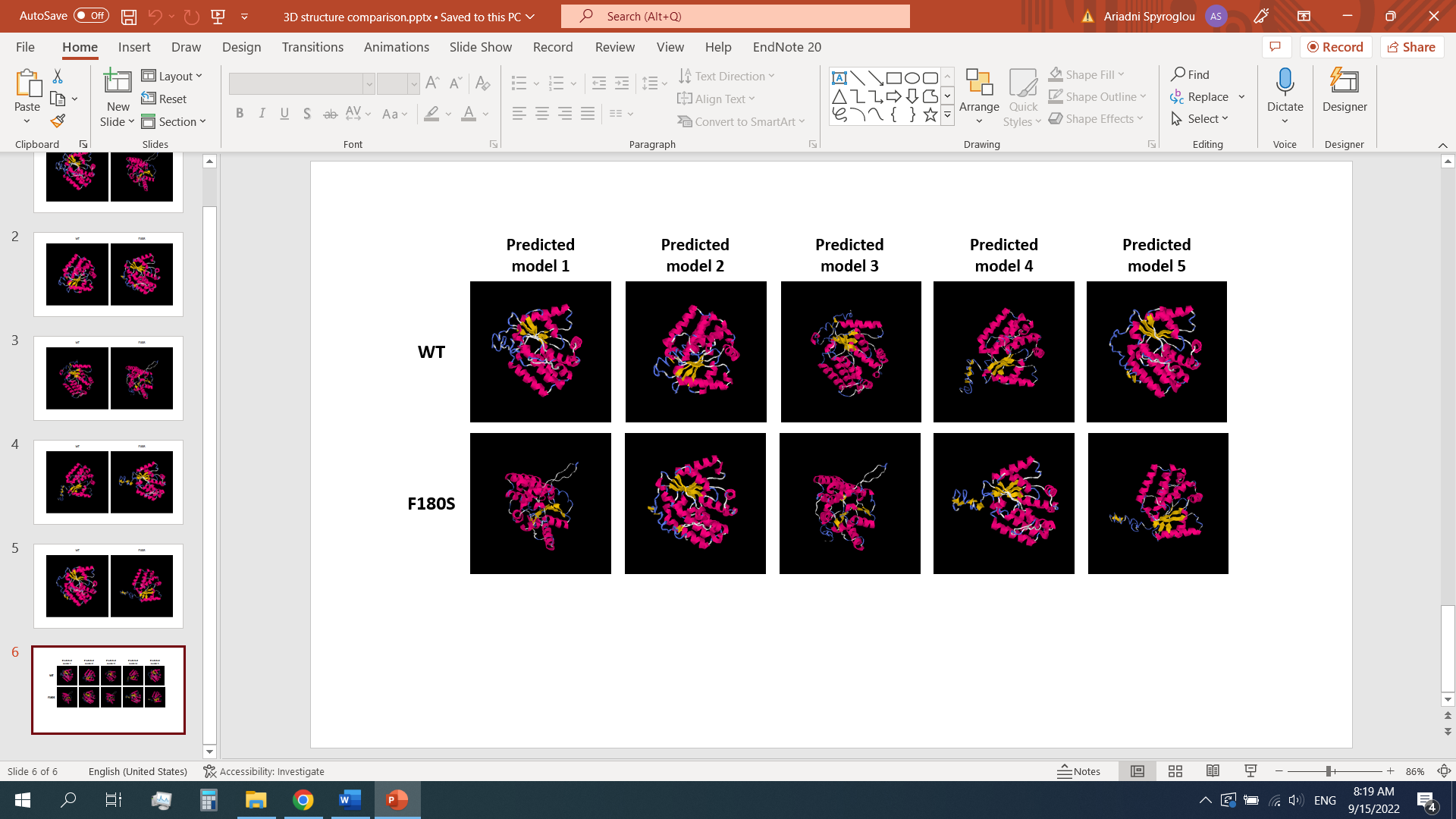


**Supplemental Table 1:** Primer pairs for sequencing or qPCR analysis of the respective genes.

| *Atp5b F* | 5’-GGTTCATCCTGCCAGAGACTA-3’ |  |
| --- | --- | --- |
| *Atp5b R* | 5’- AATCCCTCATCGAACTGGACG-3’ |  |
| *Citrate Synthase F* | 5'-CGG TTT GTC TAC CCT TCC CC-3' |  |
| *Citrate Synthase R* | 5'-GGC AGG ATG AGT TCT TGG CT-3' |  |
| *Dck F* | 5'-CAG CTA GCC TCT CTC AAT GGC A-3' |  |
| *Dck R* | 5'-GCT GTT CAT CCA GTC GTG CCA-3' |  |
| *Dguok Seq F* | 5'-GCT AAC TCT CCT GCT CTC ATG AC-3' |  |
| *Dguok Seq R* | 5'-GCT CAG AGT TAC CTG GGG TGA-3' |  |
| *Dguok c (Mut)* | TEX-gTTTgCg+gACTC+CTg--BBQ | “+” = LNA base |
| *Dguok c (WT)* | Cy5-ggTTTgCg+AAC+TC+CTg--BBQ | “+” = LNA base |
| *Dguok F Geno* | 5'-CAG TGA CAT CGA GTG GCA CA-3' | RT-qPCR genotyping |
| *Dguok R Geno* | 5'-GCT CAG AGT TAC CTG GGG TG-3' | RT-qPCR genotyping |
| *Gadph F* | 5'-GCA TTG TGG AAG GGC TCA TGA CC-3' |  |
| *Gadph R* | 5'-TTC TCC AGG CGG CAC GTC AGA T-3' |  |
| *Idh1 F* | 5'-ATG CAA GGA GAT GAA ATG ACA CG-3' |  |
| *Idh1 R* | 5'-GCA TCA CGA TTC TCT ATG CCT AA-3' |  |
| *Idh2 F* | 5’-GGAGAAGCCGGTAGTGGAGAT-3’ |  |
| *Idh2 R* | 5’-GGTCTGGTCACGGTTTGGAA-3’ |  |
| *Lep F* | 5'-ACA TTT CAC ACA CGC AGT CGG TAT-3' |  |
| *Lep R* | 5'-TGA GGA CCT GTT GAT AGA CTG CCA G-3' |  |
| *Nampt F* | 5'-GCA GAA GCC GAG TTC AAC ATC-3' |  |
| *Nampt R* | 5'-TTT TCA CGG CAT TCA AAG TAG GA-3' |  |
| *Mt-Nd1 S* | 5’-TCG ACC TGA CAG AAG GAG AAT CA-3’ | Mt-DNA quantification |
| *Mt-Nd1 AS* | 5’-GGG CCG GCT GCG TAT T-3’ | Mt-DNA quantification |
| *Nmnat1 F* | 5'-TCT TGT ACG CAT CAC CGA CC-3' |  |
| *Nmnat1 R* | 5'-GCT GGG ATG AGC CCT TTC TT-3' |  |
| *Nadsyn1 F* | 5’-ACGGCTGCTCACTACTTGTTA-3’ |  |
| *Nadsyn1 R* | 5’-CTGAGAACCGAGGCAACTTC-3’ |  |
| *Rpph F* | 5’-GGA GAG TAG TCT GAA TTG GGT TAT GAG | Genomic DNA quantification |
| *Rpph R* | 5’-CAG CAG TGC GAG TTC AAT GG-3’ | Genomic DNA quantification |
| *Sdha F* | 5'-ACT GTT ATT GCT ACT GGG GGC-3' |  |
| *Sdha R* | 5'-TAC CTG TGG GGT GGA ACT GA-3' |  |
| *Ucp1 F* | 5'-TCA GGG AGA GAA ACA CCT GCC TC-3' |  |
| *Ucp1 R* | 5'-CTT GCA TTC TGA CCT TCA CGA CCT-3' |  |
| *Tbp F* | 5'-GAA GAA CAA TCC AGA CTA GCA-3' |  |
| *Tbp R* | 5'-CCT TAT AGG GAA CTT CAC AG-3' |  |
